# Majority illusion drives the spontaneous emergence of alternative states in common-pool resource games with network-based information

**DOI:** 10.1016/j.isci.2025.112831

**Published:** 2025-06-06

**Authors:** Nicolas Schrama, Andrew R. Tilman, Vítor V. Vasconcelos

**Affiliations:** 1 Graduate School of Informatics, University of Amsterdam, Amsterdam 1090 GH, the Netherlands; 2 Computational Science Lab, Informatics Institute, University of Amsterdam, Amsterdam 1098 XH, the Netherlands; 3 USDA Forest Service, Northern Research Station, St. Paul, MN, USA; 4 POLDER, Institute for Advanced Study, University of Amsterdam, Amsterdam 1012 GC, the Netherlands

**Keywords:** Environmental science, Natural resources, Computer science

## Abstract

Common-pool resources (CPRs), including fisheries and the atmosphere, are critical for ecological, social, and economic sustainability but are easily overused. We use an agent-based model to investigate how social networks shape resource extraction outcomes. Networks with highly visible nodes can create a “majority illusion” in which most users believe high-intensity extraction is dominant, even if it is not. This misperception can push the entire population toward one of two possible states: an abundant, high-welfare resource or a depleted, low-welfare one. Aligning users’ environmental impact with their visibility can mitigate this effect and steer the system toward a single, predictable outcome. These results suggest that network-based policies, such as reshaping information flows, particularly targeting high-impact hubs, could help stabilize cooperative behaviors and encourage sustainable management of CPRs, reducing the risk of tragedy-of-the-commons scenarios.

## Highlights

• Information asymmetry generates bistable outcomes in common pool resources
• A few users drive information asymmetries in perceptions of resource extraction
• The stochastic emergence of key users’ behavior determines which outcome results
• As information asymmetries are reduced, the bistability of outcomes subsides

## Graphical abstract

**Figure:**
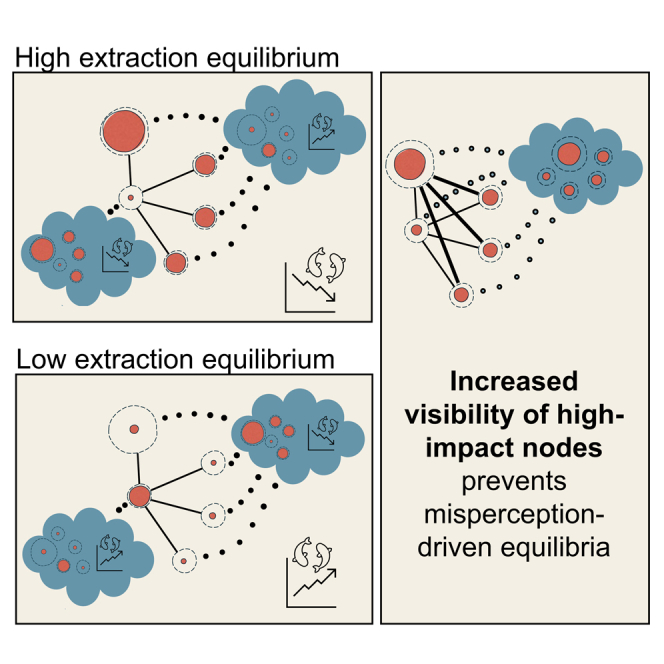

## Introduction

Common-pool resources (CPRs) are critical resources characterized by their rivalrous and non-excludable nature, presenting intricate challenges in sustainable management.^1^ Examples include certain fisheries, forests, and the atmosphere, which are foundational to ecological sustainability and human societies. The dual characteristics of rivalry and non-excludability CPRs mean that (1) one individual’s use reduces availability for others and (2) restricting access is challenging, respectively. These unique characteristics create complex resource management challenges, as traditional market mechanisms frequently fail to prevent overexploitation and degradation.^1^^,^^2^ Even so, while, e.g., many forests and fisheries are privately owned and managed—making their direct economic benefits excludable—they often provide non-excludable ecosystem services, such as carbon storage, habitat for wildlife, water quality improvements, and water quantity regulation. These ecosystem services directly impact food security, biodiversity, and climate regulation, also necessitating management and preservation.

Historically, managing CPRs has been a complex issue, often leading to scenarios described as the “tragedy of the commons”.^3^ This concept illustrates how individual users, acting independently and rationally according to their self-interest, can ultimately deplete a shared resource to the detriment of the entire group’s long-term best interests. This phenomenon is not just a theoretical construct but is evidenced in numerous real-world scenarios, such as overfishing in international waters and deforestation in the Amazon basin.^4^ The prototypical case is that of climate change mitigation, which has been framed as a collective risk dilemma,^5^ where individuals face a dilemma between investing or waiting for others to invest first to avoid damages with some probability. Theoretical and experimental evidence suggests that, beyond increasing risk perception and reducing uncertainty,^6^ communication reduces free-riding.^7^^,^^8^ Local solutions based on decentralized learning, reciprocity, and other types of formal and informal institutions have also addressed the issue.^9^^,^^10^ For instance, Ostrom’s principles of collective resource management have been instrumental in illustrating how local communities can successfully manage CPRs through collective action and self-governance.^1^ Her framework highlighted the importance of rules tailored to specific places and situations, local decision-making, monitoring, and graduated sanctions, among other factors, in effectively managing CPRs. Nonetheless, when there is a mismatch between the scale of the enforcement of the rules and that of the resources being managed, formal and informal institutions fail.^11^

Sustainable management of CPRs has been a critical area of study in environmental science and policy.^12^ However, current challenges in the area are becoming even more pronounced due to their scale and other factors like increased global demand, technological advancements in resource extraction, and climate change impacts. These challenges are further complicated by the intricate interplay of ecological dynamics and human behavior,^13^ including the increasingly sophisticated ability to anticipate future environmental states, which affects the collective ability to manage resources.^14^ Thus, various new tools are used to address the challenges posed by over-exploitation and mismanagement of CPRs.^15^^,^^16^^,^^17^

The impacts of human activities on CPRs are extensive and complex. Driven by short-term benefits, unsustainable practices can result in long-term ecological degradation and resource scarcity. These effects can have cascading implications for climate change, biodiversity, and human health.^18^^,^^19^ Systems linking people and nature, known as social-ecological systems, have been conceptualized as complex adaptive systems.^1^^,^^20^ In these systems, nonlinear feedbacks, strategic interactions, and heterogeneity pose significant challenges for effective modeling and policy-making. Ignoring these complexities can lead to ineffective or counterproductive policies.^20^^,^^21^ Drawing on complex systems science to account for the intricate interplay between ecological systems and behavioral and socio-economic factors may contribute to effective management and policy interventions, thereby supporting equitable and sustainable resource utilization that addresses diverse interests, perspectives, and stakeholders’ reach.^22^^,^^23^

Recent theoretical advances in applying evolutionary game theory to social-ecological systems have focused on the coevolution of behavior and resource dynamics, exploring how changes in human behavior can directly affect resource dynamics and vice versa.^13^^,^^24^ These advances could allow for the modeling of adaptive management strategies, where policies and practices are designed to evolve in response to changes in the system, as opposed to finding unique, optimal long-term solutions, which can be more effective in dealing with the complexities of CPRs.^25^ Adaptive management is embodied in reflective institutions that integrate continuous observations from the field into the evaluation and development of new policies.^26^

Given the ubiquity of environmental change, users of resources may try to forecast resource trends to better adapt their behaviors to changing conditions. Integrating environmental forecasting into decision-making means resource users must consider their valuation of the future. How much actors care for the future can transform the game from a tragedy of the commons into one of coordination.^27^ Additionally, under strong human-resource feedbacks that lead to variable environments (tilman evolution 2023), forecasting can foster collective intelligence by providing a public good that reduces the amplitude of environmental oscillations and often increases mean payoffs to both forecasters and non-forecasters. This coevolutionary perspective underscores the dynamic interplay between human actors and the natural environment, which is central to understanding and managing CPRs sustainably.

Despite these advancements, significant gaps remain in the literature, particularly regarding the role of information asymmetry and social network structures in CPR management. While some studies have highlighted the importance of information biases^28^ and social networks in cooperation for resource governance,^29^^,^^30^^,^^31^ the specific influence of highly visible or hub agents within these networks is less understood and has only recently gotten analytical traction.^32^ The diffusion of shared goals among resource users through networks of influence can foster cooperation and influence the sustainability of CPRs.^33^ The ability to sever ties based on strategy similarity or success influences group formation and harvesting strategies.^34^ While these findings underscore the importance of network structure and collective coordination, they generally focus on network evolution or shared incentives rather than on how heterogeneous visibility and impact within a fixed information network biases individuals’ perceptions of overall behavior. A review by Bodin and Crona shows that structural properties like density and cohesiveness can impact governance processes.^35^ Valuable case-specific empirical observations also highlight the relationship between structural aspects of social networks and resource management. In the Maine lobster fishery context, Wilson et al. demonstrate how competitive behaviors among fishers lead to the emergence of group structures that facilitate collective action for conservation.^36^ The dynamics of informal advice networks in Ghanaian cocoa agroforestry systems reveal how core farmers, often with access to external information, serve as vital conduits for transferring knowledge and practices within communities.^37^

The generalizability of these results to other contexts remains uncertain. Addressing this requires a better understanding of the information flow mechanisms among resource users and how these directly impact CPR sustainability. This gap is particularly relevant in the context of modern environmental challenges, where large heterogeneities are present—both in information availability and impact—with the actions of a few key players or institutions disproportionately affecting the sustainability of resources. Specifically, the question remains open:

How do heterogeneous degree distributions—where some individuals are highly visible and others are peripheral—systematically shape each user’s forecast of other agents’ extraction decisions and, thus, common-pool-resource outcomes?

Understanding this mechanism is crucial, as any persistent misperception or “illusion” about the majority’s actions could substantially shift strategic behavior and long-term resource dynamics and stability. This paper directly addresses that gap by demonstrating how heterogeneous information networks can create (and reinforce) majority illusions that lead to multiple stable states in CPR use.

We study how social networks influence information flow and decision-making among CPR users, focusing on the impact of influential individuals on the dynamics and stability of the resources they share. Using a game-theoretical agent-based model, it analyzes how agents’ resource extraction decisions, influenced by their social ties, co-evolve with and impact the sustainability of CPRs. We explore various cognitive parameters and information constraints within these networks, thereby providing a more nuanced understanding of CPR management under conditions of bounded rationality.

The model simulates the decision of individual agents in a population of CPR users. Agents choose between extracting from the resource with low effort or high effort, thereby influencing the evolution of the resource state. Their decision depends on their perception of the resource state and their expectations of other users’ behavior. The resource state in the model is normalized between 0 and 1. Differences in agents’ (number of) social ties generate heterogeneous information and visibility. Recognizing that highly central or visible players plausibly exert concomitant environmental impact, we consider a parameter of inequality of environmental impact that amplifies the environmental impact of agents with many social ties.

The study reveals that network structures, particularly skewed degree distributions, lead to multiple possible CPR outcomes corresponding to alternative stable states for the same external conditions. A key finding is the substantial influence of hub agents in shaping network perceptions and decisions, creating phenomena like majority illusions^38^ that significantly affect resource states and sustainability. As the inequality of impacts rises, this effect disappears, but access to a state with higher welfare also disappears. These findings highlight the critical role of network hubs in CPR dynamics, offering new directions for effective management and policy-making.

## Results

**Figure 1:**
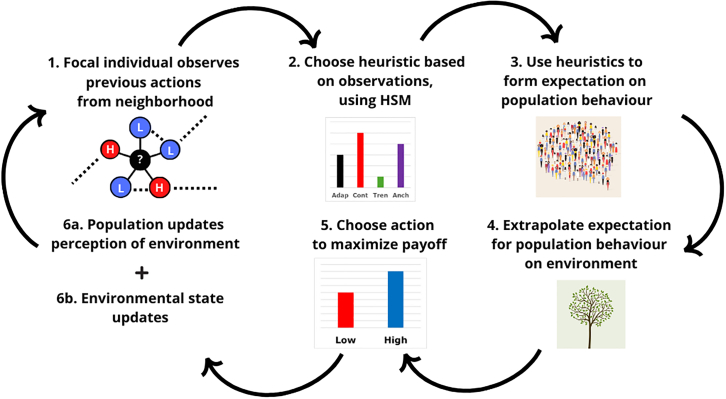
The flowchart provides a schematic representation of the steps agents’ decision making process consist of. A random agent is selected each period The probably of being selected in a round is constant at 1/P and does not depend on the number of past selections. Decision-making consists of five steps: observation, heuristic selection, forming an expectation on behavior, forming an expectation for the environment, and finally choosing an action. The schematic shows a sixth step of all agents updating their perception of the state of the environment. The STAR Methods provide more detail on these subprocesses.

The model can simulate diverse CPR scenarios that differ in terms of environmental dynamics, user incentives, and social network structures. Individuals learn to use different heuristics about the future state of the environment using the information they have, based on the heuristics switching model, to decide between a high and low effort extraction.^39^ In the STAR Methods section, we detail how individuals generate expectations about others’ behavior. We further provide a description of how this model results in previously published and analytically tractable models under the assumption of full information and that the expectations about future behavior are the same as the current behavior. Figure 1 summarizes agents’ decision-making process. In this paper, we study a CPR with two crucial characteristics.

First, we consider cases where individuals have an incentive to switch to the low-effort strategy when the environment is at its most depleted state and an incentive to switch to the high effort strategy when the environment is at its most bountiful state. The environmentally dependent payoff matrix,

(Equation 1) $$\Pi \left(n\right)=\underset{\text{weight}}{\underset{︸}{\left(1-n\right)}}\underset{\text{degraded}\;\text{environment}\;\text{payofffs}}{\underset{︸}{\left[\begin{array}{cc} R_{0} & S_{0}\\ T_{0} & P_{0} \end{array}\right]}}+\underset{\text{weight}}{\underset{︸}{n}}\underset{\text{prosperous}\;\text{environment}\;\text{payoffs}}{\underset{︸}{\left[\begin{array}{cc} R_{1} & S_{1}\\ T_{1} & P_{1} \end{array}\right]}}\text{,}$$

describes the payoffs of the four types of interactions. The first row corresponds to the low-effort strategy, and the second row corresponds to the high-effort strategy. The first column corresponds to the payoffs derived when others are extracting with low effort and the second when they extract with high. For instance, a low-effort individual in a degraded environment (n=0), when interacting with other individuals with low effort, will receive a payoff $R_{0}$, whereas a high-effort behavior interacting with other high-effort individuals in a prosperous environment (n=1) will get $P_{1}$. We can describe the collective incentives to switch strategies in terms of differences in payoffs when n=1 and when n=0.^13^ We define the difference $S_{0}-P_{0}$ as the payoff advantage of being an early adopter of the low effort strategy in a depleted environment, the difference $R_{0}-T_{0}$ as the payoff advantage of being a late adopter of low effort in a depleted environment, the difference $T_{1}-R_{1}$ as the payoff advantage of being an early adopter of high effort in a bountiful environment, and the difference $P_{1}-S_{1}$ as the payoff advantage of being a late adopter of high effort in a bountiful environment following the approach of Tilman et al.^13^ Capital Δ’s denote the incentives to switch to the high-effort strategy, and lowercase *δ*’s denote the incentives to switch to the low-effort strategy. Superscripts correspond to the current environmental state, and subscripts denote the current strategy followed by the rest of the population.

In the main text, we let $S_{0}-P_{0}>0$ and $T_{1}-R_{1}>0$, creating incentives against coordination. These incentives reflect cases where opportunistic extraction (choosing high effort when others opt for low effort) is rewarded when the resource is bountiful, yet there are positive incentives for early adopters of an “environmentally friendly” low effort strategy when the resource is depleted.

The concept of coordination incentives is closely related to expectations feedback in the learning to forecast literature.^40^^,^^41^^,^^42^ Research in this domain highlights that incentives to act against the majority lead to stable dynamics that tend to the full-information equilibrium (FIE), which is defined as the equilibrium state that would be attained under a perfect-information, well-mixed version of the model. Conversely, incentives favoring coordination with the majority can result in oscillating dynamics that fail to converge to the FIE.

The second crucial characteristic of the research setting is that social network structures are generated using the Barabasi-Albert algorithm, which results in skewed degree distributions and tends to scale-free networks as the number of individuals increases. Table parameter values provides the complete set of default parameters, which we use throughout unless specified otherwise.

### A majority illusion creates bistable resource outcomes

**Figure 2:**
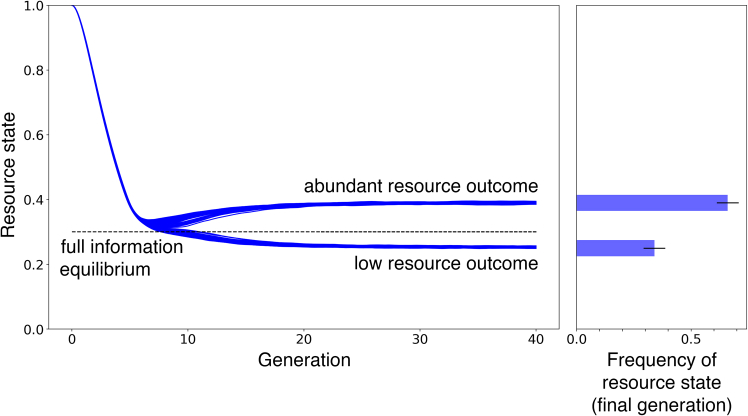
Information asymmetry generates two alternative equilibrium levels of resource outcomes The system spontaneously evolves into a noise-sensitive region, where microscopic differences—i.e., precisely which sets of agents is doing what—leads the system into one of two alternative states. Here, asymmetries among agents are created by the social network, a Barabasi-Albert network with scale-free distribution of links, which defines the differences in how much information agents obtain and which information they obtain. Agents that obtain more information are also visible to more agents of the rest of the population. In this setup, the model’s incentive structure encourages agents to go against the majority. Each blue curve represents the resource level over time for a single simulation on a heterogeneous network. The dashed line indicates the full-information equilibrium. The right panel shows the distribution of the model outcomes. There is a 35–65 split of the simulations, with about 35% resulting in a stable resource level below the full-information equilibrium, FIE (dashed lined), and 65% above. This plot shows the results of 100 simulations of the model that differ in seed, affecting their specific network connections and the stochasticity they experience.

Figure 2 illustrates the results of many simulations of the model for a single parametrization. The simulation results reveal that the system is at first attracted to an equilibrium close to the FIE. This region, however, is noise sensitive and gives access to two alternative states. The different simulations end up splitting into two different paths with alternative equilibrium levels of the resource: an abundant outcome and a scarce outcome.

The right panel of Figure 2 shows the frequency of the two outcomes, demonstrating a 35–65 split, with about 35% of the simulations resulting in a resource level below the FIE. These findings prompt the question of why two possible resource outcomes arise and what dynamics, or microscopic variation, underlie deviations from the FIE and the stochastic switching that occurs near it.

**Figure 3:**
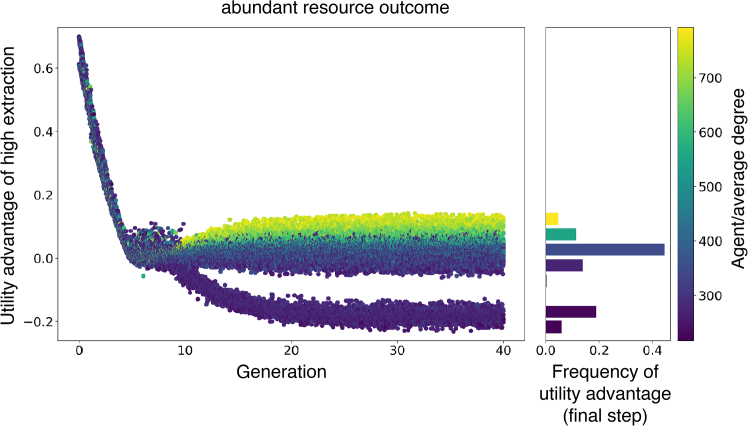
Distribution of the perceived utility advantage of high-effort extraction relative to low-effort extraction in a single typical abundant resource state simulation, corresponding to an abundant resource outcome The utility advantage is what drives behavior. Each dot corresponds to a single agent in a single simulation round. The color of the dot represents that agent’s degree. Agents displayed in yellow have many social ties, while the agents displayed in dark purple have few social ties, as indicated by the label. The histogram on the right side provides the final distribution of the utility advantages. The colors of the bars represent the average degree of the agents that perceived these (binned) utility advantages. The frequencies in the bar plot are based on the last 10000 time points over 100 simulations. In Barabasi-Albert networks, a few agents possess most of the connections. The abundant outcome occurs when the network’s most connected agents extract with high effort. The visibility of these agents creates the perception among most agents that the average extraction level in the population is high. The phenomenon where globally rare network states that are overrepresented in the local neighborhoods of many individuals is called the majority illusion. As a result of incentives to go against the (perceived) majority, the predominant share of the population, which has few connections, extracts with low effort, shown here as the lower branch in the left matching the modal peak on the distribution of the right. Consequently, the realized population-level extraction is low, leading to an abundant resource state.

To look into the microscopic details, we delve into the incentives agents face over time to answer these questions in each of the two possible (high and low resource abundance) outcomes. Figure 3 illustrates the utility advantage of high effort extraction, $\pi_{H}-\pi_{L}$, perceived by individuals over time from a single simulation that results for the abundant resource outcomes (corresponding to the top lines in Figure 3).

Figure 3 demonstrates that agents’ perception of the utility advantage of high effort, at first, follows a narrow band, with agents agreeing on the marginals of the different high- and low-extraction options. As the system nears the FIE, fluctuations increase and there is a spontaneous sorting in terms of agents’ degree, where the network’s most connected agents tend to perceive the highest utility advantage of high effort. In contrast, the least connected agents perceive a utility advantage for low effort. This sorting of agents by degree is depicted by the colors of the dots in the left panel and the bars in the right panel. Highly connected agents, perceiving exclusively positive utility advantages, consistently opt for high-effort extraction. The visibility of these agents creates the perception among most users that the average extraction level in the population is high. The phenomenon where globally rare network states are over-represented in the local neighborhoods of many individuals is called the majority illusion.^38^ Due to incentives against coordination (to act against the majority), the predominant share of the population chooses low-effort extraction. Consequently, the realized population-level extraction is low, resulting in an abundant resource state. Thus, in systems with incentives to oppose the majority, beneficial resource outcomes emerge when highly visible users extract from the resource extensively, thereby incentivizing others to sustain it.

**Figure 4:**
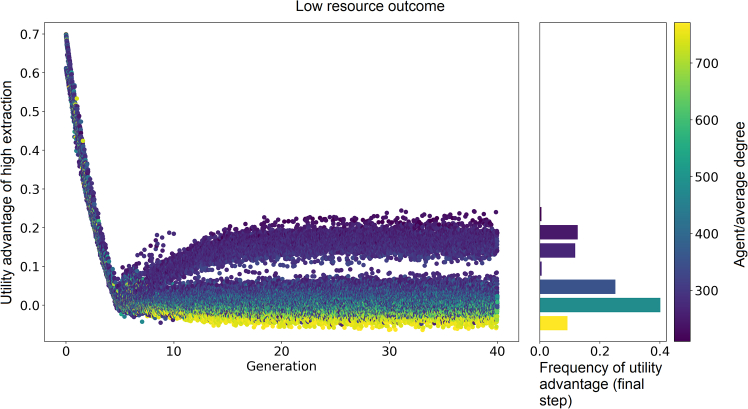
Distribution of the utility advantage of high-effort extraction relative to low-effort extraction in a single typical low resource outcome simulation A scarce resource state emerges when the network’s most connected agents extract with low effort because the most connected agents perceive a negative payoff advantage to high-effort extraction. This creates a perception among a majority of agents of low population-level extraction. This perception results in an incentive to extract with high effort among agents with few connections and, in turn, a scarce resource outcome. In other words, a relation exists between state and degree. When high-degree agents extract with high effort, i.e., a positive correlation between state and degree, the abundant outcome emerges, while a negative correlation leads to a scarce outcome. This correlation arises when the utility advantage reaches zero, and the first users start to extract with low effort. The sign of the correlation settles randomly within a few generations after its manifestation and persists throughout the simulation. Thus, the state-degree correlation and its stochastic emergence explain the bistability in the model’s steady states.

Figure 4 shows the utility advantage over time in an illustrative simulation that results in a scarce resource state (corresponding to the bottom lines in Figure 2). Like in Figure 3, the initially consensus on the marginal utilities reaches a region of high variance near the FIE. In contrast to the setups that end in high resource levels, the assortment of agents’ payoff perceptions and network degree in Figure 4 is reversed, as the most connected users now perceive the low-impact strategy as preferable in the long run. This results in scarce resource outcomes because when the network’s most connected users opt for low-effort extraction, it creates a misperception among many agents of low population-level extraction. Consequently, most users (who have mostly few connections) are incentivized to engage in high-effort extraction, resulting in a scarcer resource outcome.

Figures 3 and 4 highlight the relationship between users’ perceptions and their degrees. When higher-degree users are more likely to choose to extract with high effort—a positive correlation between effort and degree—the abundant outcome emerges, while a negative correlation leads to a scarce outcome. This correlation emerges after a transient period where the utility advantage approaches zero. Then the first users start to switch their strategies and the sign of the correlation between degree and effort arises randomly within a few generations and persists throughout the simulation. Thus, the emergent effort-degree correlation and its stochastic emergence explain the bistability observed in the model’s equilibria.

With this analysis of the behavior of individuals across groups of with degrees, we are now in a position to further understand the results of Figure 2. Since the symmetric break occurs when individuals reach their indecision point (no utility advantage) and the resource state reaches the FIE, it can be explained starting from the model’s equilibrium mechanics. The overall prevalence of the abundant equilibrium (fraction of simulations that result in a state more abundant than expected by the FIE) depends on the environment’s critical level. The environment’s critical level is the level of the environment where the utility advantage equals zero, given population behavior. By the definition of the environmental update equation (see Equation 11 in the STAR Methods), a FIE equilibrium at, say, n=0.3, requires 70% of all users to extract with high effort. The lower the environmental state in equilibrium, the higher the fraction of users extracting with high effort needed. Therefore, when the environment reaches its critical level, the probability of (a majority of) hub users (who are a minority group) extracting with high effort is greater than not, and when the corresponding FIE of the environment is lower the probability that hub users are in high extraction state is higher. As a result, high-effort extraction is overrepresented more frequently, leading the majority of users to under extract. This results in a higher prevalence of the abundant outcome. This outcome is independent of the initial conditions of the resource and distribution of low effort extractors.

### The skewness of the degree distribution and alternative stable resource states

The structure of the social network is critical for these correlations to build up. In particular, the skewness of the degree distribution is a critical factor in determining the existence of alternative stable resource states and the magnitude of divergence between these states.

We consider networks generated by the Barabasi-Albert algorithm and vary the skewness of their degree distribution. We use the standard algorithm, which operates by sequentially adding new nodes and connecting them to existing ones proportionally to their degree. The algorithm’s parameters are the number of nodes at termination (*P*) and the number of existing nodes to which a new node is connected (*λ*). A high value of *λ* yields a significantly skewed degree distribution.^43^

**Figure 5:**
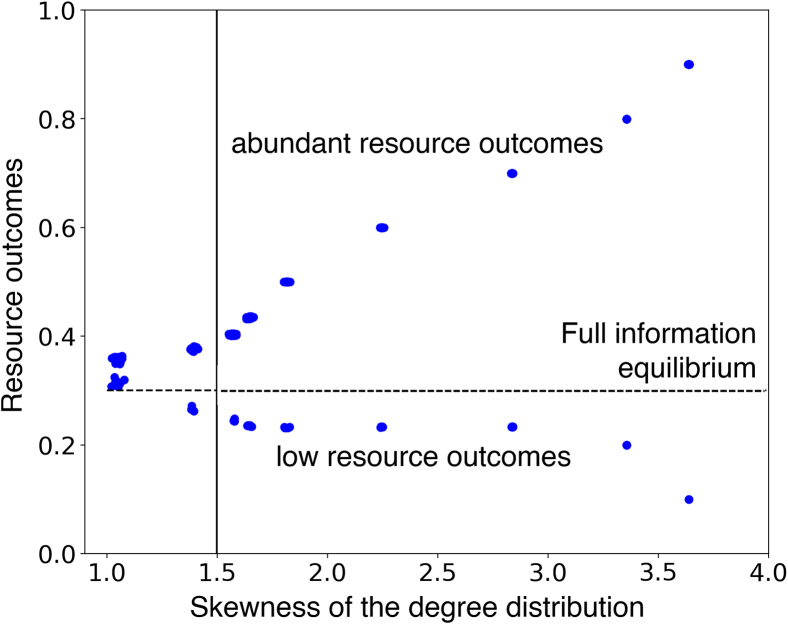
Spread of the model outcomes increases as the skewness of the degree distribution increases This skewness results from the initialization of the BA algorithm. For example, if the network has 1000 nodes and 950 nodes are added for each new node, the network is initialized with 950 nodes with no edges. Then, 50 nodes are added sequentially with 950 connections each. These become the hubs. The other 950 nodes end up with about 48–50 connections. This results in a skewed degree distribution. Each dot corresponds to the average resource state in the last hundred time steps of a simulation. The plot is based on 30 simulations per skewness level. The dashed horizontal line represents the FIE, while the solid vertical line corresponds to the skewness level of the degree distribution in Figures 2, 3, and 4. The plot shows the emergence and persistence of bistability in the model as the skewness increases. The difference between the steady states for a given link density increases as the skewness increases. As the degree distribution becomes more skewed, two things occur: (1) the middle of the degree distribution disappears while the number of low-degree agents increases, and (2) the degrees of most agents decrease. Consequently, the low-degree agents’ perception of population extraction levels becomes more skewed toward the hubs. Incentives to go against the majority drive this majority of agents to choose high effort when the hubs choose low effort and vice versa. Because of the increasing size of this group, the difference between the two steady states increases.

Figure 5 displays long-run resource state outcomes across varying skewness levels of the degree distribution. It illustrates the emergence and persistence of bistability in resource state outcomes as the skewness of the degree distribution increases. For unskewed distributions, like an Erdös-Renyi, bistability does not occur.

The difference between the resource steady states for a given link density increases as the skewness increases. As the degree distribution becomes more skewed, two things occur: (1) the middle of the degree distribution disappears while the number of low-degree agents increases, and (2) the degrees of most agents decrease. Consequently, the perception of population extraction levels by low-degree agents, who form the majority of agents, becomes more skewed toward the hubs. Incentives to go against the perceived majority drive this majority of agents to choose high effort when the hubs choose low effort and vice versa. Because of the increasing size of this group, the difference between the two steady states increases.

### Inequality of environmental impact mitigates the majority illusion

As we showed, the heightened visibility of hubs in significantly skewed degree distributions results in a more pronounced difference between the model’s two stable resource outcomes. However, highly connected agents may also vary in other ways from agents with few connections. In this section, we explore impact inequality, where different agents have different magnitudes of environmental impact, and we explore cases where there is a positive correlation between the network degree of an agent and their ability to impact the environment. This could arise when CPR users with substantial visibility also exert concomitant environmental impact. To address this relationship and potential effect, we introduce a parameter to control the strength of inequality of environmental impact (*ν*), which allocates a greater impact on the environment to highly connected individuals when ν>0.

A user’s environmental impact is determined by an exponent in the network degree that determines the (inequality of) environmental impact. As the inequality of environmental impact increases, the evolution of the resource state becomes increasingly dependent on high-degree users and less on low-degree users. When the environmental impact inequality parameter is zero, the environmental impact of all agents is homogeneous, as in all previous figures.

**Figure 6:**
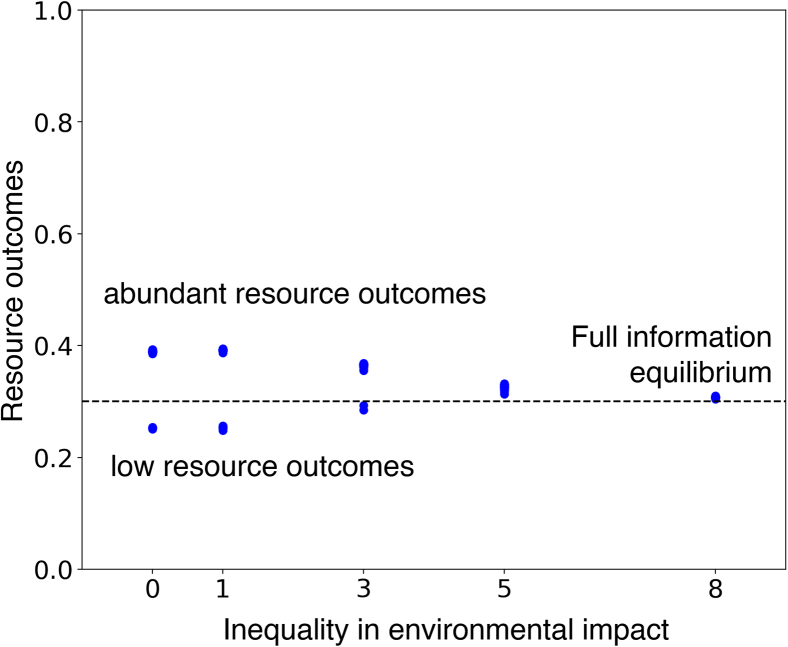
Bistability disappears when high-degree nodes also have a disproportionate environmental impact When the inequality of impact increases, the evolution of the resource state becomes more dependent on high-degree agents and less dependent on low-degree agents. The horizontal axis corresponds to the magnitude of inequality of environmental impact. The vertical axis displays long-run environmental steady states. Each dot corresponds to the average resource state in the last hundred time points of a simulation. The figure is based on 30 simulations per value of the environmental impact parameter, and the dashed horizontal line indicates the FIE. The plot shows the subsidence of bistability in the model as the inequality of environmental impact increases. Agents’ environmental impact is homogeneous when the inequality of the environmental impact parameter equals zero, as in Figure 2. As inequality of environmental impact increases, the difference between the model’s two steady states decreases, leading to the subsidence of bistability when inequality of environmental impact reaches a critical value. This single steady state equals the FIE. The deviations from the FIE stem from the overrepresentation of high-degree agents’ decisions in the local neighborhoods of many agents, creating a majority illusion. As inequality of environmental impact increases, agents’ perception of the population extraction level becomes more in line with the true extraction level because high-degree are not only overrepresented in the local neighborhoods of low-degree agents but also in terms of their environmental impact. Consequently, low-degree agents’ information becomes more accurate as inequality of environmental impact increases, steering the model outcome toward the FIE. Thus, bistability exists in the model when inequality of environmental impact is below its critical value, while there is a single steady state at the FIE when it’s at or above its critical value.

Now, we focus on how the distribution of the model outcomes changes as agents’ environmental impact becomes increasingly related to their degrees. Figure 6 displays resource state outcomes across various levels of inequality of environmental impact.

Figure 6 illustrates the diminishing difference between the model’s two steady states as the inequality of environmental impact increases, culminating in the subsidence of bistability when the inequality of environmental impact reaches a critical value. The single remaining steady-state aligns with the FIE.

The deviations from the FIE in Figure 5 stem from the overrepresentation of high-degree users’ behavior in the local neighborhoods of many users, creating a majority illusion. As the inequality of environmental impact increases, users’ perceptions of the population extraction level become more aligned with the true extraction level. This alignment occurs because hubs are overrepresented not only in terms of visibility but also in terms of environmental impact. Consequently, low-degree users’ information becomes more accurate as the inequality of environmental impact increases, guiding the model outcome toward the FIE.

Bistability is detected in the model when the inequality of environmental impact is below its critical value, whereas a single steady state exists at the FIE when the inequality of environmental impact is at or above its critical value.

The consequences of increasing inequality in environmental impact are, thus, 2-fold. On the one hand, the stable value for the resource in the equilibrium with high availability of resources is reduced (and the value for the low equilibrium increases). On the other, because the influence of high-degree agents on the environment is greater, the fact that these agents are more visible no longer results in a majority illusion effect because these agents do actually exert the majority of the impact on the environment.

## Discussion

CPR users operate in environments where the strategies of others are not always common knowledge. If access to information is incomplete and unequal, more connected individuals may have better information and exert greater influence on strategy dynamics. We investigate the impact of social networks in the context of CPR use, highlighting the role of information asymmetries and strategic behavior, particularly among hub users. We demonstrate that network structure substantially impacts resource dynamics, generating two stable environmental states. This bistability yields access to a high resource equilibrium above the FIE, and the risk of ending up in a low-resource state. The high (and low) environmental states are stabilized as the consequence of a majority illusion where highly connected agents bias the perceptions at the population level. The difference in resource states between these two outcomes depends on the balance between hub-users’ visibility and their relative environmental impact. Heterogeneity is not a necessary requirement for this bistability of the resource. Other research, considering more complex dynamics of the environment, including Allee effects, also finds bistable resource outcomes, even with homogeneous agents.^44^

The present work builds on tractable mathematical models and real-world case studies. Compared to existing tractable models of eco-evolutionary games, (13; tilman_evolution_2023; 27), the present study offers a behavioral model with heuristic-based decision-making and network-based information sets. This complexity adds realism to this class of models. In comparison to applied studies, such as^36^^,^^37^ the use of an agent-based model provides a systematic framework for exploring different decision-making processes, complex resource management scenarios, user incentives, and social network structures.^45^ As a result, we take the observation that social networks exist among resource users and come to a comprehensive explanation of how network structures impact CPR sustainability.

In the context of Ostrom’s principles, social norms, local monitoring, and reciprocity play a crucial role in stabilizing cooperation and can be seen as mechanisms allowing for multi-stability in CPR sustainability. The majority illusion presented here can disrupt or reinforce these dynamics. If highly connected actors appear to exploit resources aggressively, they may create the illusion that defection is widespread, eroding trust and reciprocity, thereby shifting the system toward unsustainable equilibria. Conversely, if hubs model cooperative behavior, they can amplify norm compliance, reinforcing rule enforcement and stabilizing sustainable outcomes. This misperception-induced bistability complements and counterbalances the mechanism we present in this paper, which sustains cooperation by aligning behavior with ecological constraints.

This study underscores the importance of considering social network dynamics and information asymmetries in designing effective environmental management policies. Our analysis showed that the relationship between environmental impact inequality and social network connectedness shaped how information asymmetries manifest as alternative stable environmental states. When hub agents have a disproportional influence on the dynamics of the environment, the potential for a “majority illusion” to result in alternative stable states was mitigated. This may offer valuable lessons for policymakers aiming to enhance resource governance. There may exist cases where successful high resource levels rely on biased information, which will likely lead to unintended consequences. Our results suggest that managers may benefit from first investigating the system’s incentives, the structure of the social network, and whether visibility and environmental impact are aligned. When the rules of the game incentivize early adoption of strategy change at high and low resource states, managers may be able to improve environmental outcomes by reconfiguring how information is shared among resource users so that the actions of those with substantial environmental impact are more visible. Policymakers can leverage these findings to develop more adaptive and informed approaches to management, fostering sustainability, such as in considering the multifaceted role of large corporations and how they may skew the perceived incentives for others.^46^

For instance, influential fishing companies with extensive networks and resources can be seen as “hub agents”.^47^ These entities often have access to better technology and information regarding fish populations and migration patterns, allowing them to anticipate fish stocks and significantly influence fishing practices and sustainability efforts.^48^ Large agricultural businesses may also act as hub agents, with significant influence over water use practices. These businesses often have more sophisticated water extraction and monitoring technologies, giving them a competitive advantage and a greater influence on groundwater levels.^49^ The Amazon rainforest’s deforestation crisis highlights the role of influential corporations and landowners as hub agents in exploiting forest resources; these agents often have extensive social and political networks, enabling them to shape land-use policies and practices.^50^ Urban air quality management, particularly in megacities, involves managing the CPR of clean air. In these settings, some industries can be seen as hub agents due to their significant contributions to air pollution but also drivers of “best practices” that permeate their respective sectors.^51^

The study finds that when hub agents have disproportionate influence it can mitigate the potential of the majority illusion to cause the alternative stable states. This finding arises because of the alignment of visibility and environmental impact and could inform strategies for more equitable and sustainable management and reduced uncertainty on environmental outcomes. We notice that the effect of biased information is not always negative since there is a stable state above the FIE. Not addressing this information bias or even deliberately withholding information to promote better environmental outcomes, however, may have other unintended consequences. Besides ethical concerns, covert nudges offer limited scope for securing lasting behavior change.^52^ Moreover, lack of transparency can erode public trust in policymakers and reduce compliance with future policies.^53^

In the context of these findings, it is advisable to counteract the information asymmetries that are the foundation for majority illusions. Policymakers should attempt providing full information to all users, or ensuring that visibility is aligned with extraction levels. An effective toolbox might consist of local bridging institutions that identify key users and share their activities publicly. A key aspect of an effective bridging institution is its visibility. A core task of these institutions must therefore be promoting their own activities among users.

For instance, policies that increase transparency and information sharing about fish stocks and sustainable practices could help align the actions of smaller, less connected fishers with sustainability goals. Encouraging the adoption of water-saving technologies and practices among all users, facilitated by knowledge transfer from hub agents, could mitigate the risk of groundwater depletion. Creating incentives for sustainable land use and strengthening the enforcement of environmental regulations, namely regarding the visibility of effective resource extraction, could help balance the interests of different stakeholders and promote rainforest conservation. Implementing stricter emissions standards and promoting green technologies could help mitigate the majority illusion effect, where the actions of a few lead to a perception of high pollution levels, discouraging collective action for cleaner air.

To facilitate managers in comprehending the influence of social network structures on CPR outcomes and creating effective policies, we developed a publicly available visual tool.^54^ The tool empowers managers to analyze network structures, identify key influencers, and evaluate potential policy interventions by offering a user-friendly interface with customizable options. We invite managers and practitioners to use the tool and to provide valuable feedback to enhance its functionality.

Future research endeavors could explore the transferability of the study’s findings to diverse CPR contexts, shedding light on the interaction between social network structure and case-specific factors. This undertaking holds significant potential for putting the study’s results into practical use and informing policy. By examining the applicability of the study’s findings across different contexts, future research could contribute to developing models for more contextually relevant and effective CPR management.

### Limitations of the study

In this study, we have focused on a specific property of information asymmetry, the skewness of the degree distribution. While the Barabási-Albert network is a convenient model for heterogeneous degree distributions, it is somewhat an extreme example. Real CPR user networks may exhibit clustering or community structures. Future work could explore these topologies to see if majority-illusion dynamics persist and or propagate among communities. Additional topological features may be relevant in specific contexts. Indeed, the model’s abstraction simplifies the specifics of real-world CPR management, limiting insights into the specific leverage points to shape the interaction between hubs’ influence on network perceptions and decisions with context-specific factors, such as social norms, the spatial distribution of the resource, or local laws.^9^ Immediate applicability to practical CPR management contexts requires mapping the model components to context-specific factors and adding new elements. To study the effects of potential policies, managers could formulate these policies in terms of the constructs present within the model, which may be limiting. Nonetheless, the co-design of system specificities and, importantly, management-specific interventions can leverage the tools provided here for rapid development.

## Resource availability

### Lead contact

Requests for further information and resources should be directed to and will be fulfilled by the lead contact, Vítor V. Vasconcelos. Additionally, Nicolas Schrama is also a corresponding author (v.v.vasconcelos@uva.nl and npn98@hotmail.nl).

### Materials availability

This study did not generate new materials.

### Data and code availability

• The article does not use pre-existing data.
• All original code used for this article is available at Zenodo under the https://doi.org/10.5281/zenodo.10657828,^55^ including the graphical user interface available under the https://doi.org/10.5281/zenodo.10566362.^54^ Both are publicly available as of the date of publication.
• Any additional information required to reanalyze the data reported in this paper is available from the lead contact upon request.

## Acknowledgments

The findings and conclusions in this publication are those of the authors and should not be construed to represent any official USDA or U.S. Government determination or policy. This work was supported by the 10.13039/100006959USDA Forest Service (Forest Service agreement number 22-IJ-11242309-044) and the Computational Science Lab, Informatics Institute, 10.13039/501100001827University of Amsterdam. A.R.T. acknowledges support from 10.13039/501100001862Formas (grant no. 2020-00454). V.V.V. acknowledges Casper van Elteren for helping with the setup of the distributed calculations.

## Author contributions

Conceptualization, A.R.T. and V.V.V.; model implementation, N.S.; model analysis, N.S., A.R.T., and V.V.V.; writing – original draft, N.S.; writing – review and editing, N.S., A.R.T., and V.V.V.; funding acquisition, A.R.T. and V.V.V.; supervision, V.V.V.

## Declaration of interests

Nicolas Schrama is also affiliated with the Dutch Ministry of Finance. This affiliation does not lead to any competing interests.

## STAR★Methods

### Key resources table

**Table:** 

REAGENT or RESOURCE	SOURCE	IDENTIFIER
**Deposited data**		

1.0	https://github.com/bobbyleg/Centrality_and_environmental_impact/	N/A

**Software and algorithms**		

Source Code	https://doi.org/10.5281/zenodo.10657828	N/A
GUI Code	https://doi.org/10.5281/zenodo.10566362	N/A
1.24.3	Numpy	N/A
3.7.2	Matplotlib	N/A
2.0.3	Pandas	N/A
3.1	Networkx	N/A
4.65	tqdm	N/A

### Method details

#### Agent-based model

A complete, detailed model description, following the ODD (Overview, Design concepts, Details) protocol^56^^,^^57^^,^^58^ is provided in the GitHub repository with the code and the associated static Zenodo.^55^ The visualization tool is also static.^54^

This paper aims to study the role of information access on strategic behaviors of CPR users through social networks. Agents choose between extracting from the resource with low effort or high effort, thereby influencing the evolution of the resource state. Differences in agents’ number of social ties generates heterogeneous visibility. Recognizing that highly visible players can potentially have a large environmental impact, we consider an inequality of environmental impact parameter that amplifies the environmental impact of agents with many social ties.

The model includes the following entities.

• agents that represent common-pool resource users and
• the environment.

The state variables characterizing these entities are listed in the Table State variables of the model:

**Table:** State variables of the model

Entity	State Variable	Relevance
Agent	Memory ledger	Tracks other agents’ strategic decisions, used for
		observation and resource state perception updating
	Observations	Snapshots of population-wide extraction levels used
		to form expectations about future extraction levels
	Resource state perception	Used for choosing a strategy
	Heuristic	Rule used for expectation formation
Environment	Resource state	Model output of interest

The model runs for *P* agents, with P=1000 in the figures, for T=40000 time steps or 40 generations, enough for the model to reach a steady state.

The processes that repeat every time step are:

• the random selection of an agent,
• four processes concerning the selected agents’ decision-making (observation, heuristic selection, expectation formation, and payoff evaluation),
• the updating of the average extraction level,
• the updating of the resource state, and
• the updating of all agents’ perceptions of the resource state.

To avoid order and synchronicity effects, at each time-step, an agent is randomly selected to act, approximating a continuous time model behavior.

The most important design concepts of the behavioral model are objectives, prediction, and sensing.

Utility maximization, which occurs according to the payoff evaluation submodel, is the main objective. It drives agents’ adaptive behavior and, in turn, the evolution of the resource.

Given the computational and information constraints implied by bounded rationality, making an optimal decision requires agents to form expectations about strategic behavior in the population. This process is based on the Heuristics Switching Model^39^ which offers a menu of simple rules that imply realistic computational costs.

Moreover, agents with more information about the game they are playing can make better decisions. Sensing entails acquiring information on the strategic behaviors of the other players and is carried out by the observation submodel.

Key processes in the model are observation, expectation formation, payoff evaluation, updating the average extraction level, and updating the resource state.

#### Observation of peers

Agents observe the strategic decisions of all users with whom they have a social tie. Social ties determine the network of information.

• **Social Network Connections**: Each agent is connected to others through a social network generated using the Barabási-Albert algorithm, which produces a scale-free network with a skewed degree distribution.
• **Memory Ledger**: Agents maintain a memory ledger that records the most recent strategies (L or H) of their connected peers (neighbors).
• **Information Access**: Agents can only observe and update their ledger with the actions of their direct connections, reflecting the limited information access inherent in real-world social networks.

##### Perception of resource state

Agents update their perception of the resource state each round. They take a snapshot of their ledger whenever they act and use these snapshots to form expectations.

• **Local Observations**: Agents use their memory ledger to form a perception of the current resource state by averaging the extraction efforts of their observed peers.
• **Perception Updating**: Each agent updates their perception of the resource state ($n_{t-1}$) in every period using the resource dynamics equation (see Equation 11) and their local observations.

#### Expectation formation

Agents form expectations about the future extraction levels of the population ($z_{t}^{e}$) using heuristics from the Heuristics Switching Model (HSM).^39^ The HSM provides a menu of simple, boundedly rational rules that agents can switch between based on their past performance.

• Available Heuristics:
  1. Adaptive Expectations:
    $$z_{t}^{e}=\beta_{1}z_{t-1}+\left(1-\beta_{1}\right)z_{t-2}^{e}$$
  2. Trend-Chasing Expectations:
    $$z_{t}^{e}=z_{t-1}+\beta_{2}\left(z_{t-1}-z_{t-2}\right)$$
  3. Contrarian Expectations:
    $$z_{t}^{e}=z_{t-1}+\beta_{3}\left(z_{t-1}-z_{t-2}\right)$$
    *Note*: $\beta_{3}$ is negative for contrarian behavior.
  4. Anchoring-and-Adjustment:
    $$z_{t}^{e}=0.5\left(z_{\text{ave}}+z_{t-1}\right)+\left(z_{t-1}-z_{t-2}\right)$$
    where $z_{\text{ave}}$ is the average of past observations.

#### Heuristic selection and updating

Agents periodically update their choice of heuristic based on the historical performance of each heuristic in predicting others’ behaviors.

• **Performance Evaluation**: The performance of each heuristic is assessed using a fitness function based on the squared forecasting error:

$$f_{h,t-1}=-{\left(z_{t-1}-z_{h,t-1}^{e}\right)}^{2}+\eta f_{h,t-2}$$

• where:
  ○ $f_{h,t-1}$ is the fitness of heuristic *h* at time t−1.
  ○ *η* is the memory parameter (0≤η≤1) representing the weight of past performance.
• **Probability of Heuristic Selection**: The probability that an agent selects heuristic *h* is given by a discrete choice model:

$$P_{i,t}\left(h\right)=\rho \;h_{i,t-1}+\left(1-\rho \right)\frac{\;\exp\;\left(\phi \;f_{h,t-1}\right)}{\sum_{j}\;\exp\;\left(\phi \;f_{j,t-1}\right)}$$

• where:
  ○ *ρ* is the inertia parameter representing the probability of retaining the current heuristic.
  ○ *ϕ* is the choice intensity parameter reflecting sensitivity to performance differences.
  ○ $h_{i,t-1}$ indicates whether agent *i* used heuristic *h* at time t−1.

#### Payoff evaluation

Payoff evaluation is performed by computing the advantage of extracting with low relative to high effort. The payoffs depend on an agent’s strategy relative to the population and the resource state. Starting from an environmentally dependent payoff matrix

(Equation 2) $$\Pi \left(n_{t-1}\right)=\left(1-n_{t-1}\right)\left[\begin{array}{cc} R_{0} & S_{0}\\ T_{0} & P_{0} \end{array}\right]+n_{t-1}\left[\begin{array}{cc} R_{1} & S_{1}\\ T_{1} & P_{1} \end{array}\right]$$

we construct the payoff functions for low- and high-effort resource extraction. These payoffs drive agents’ adaptive behavior and depend on the environmental state ($n_{t-1}$) and expectations regarding the other users’ strategic decisions ($z_{t}^{e}$):

(Equation 3) $$\begin{array}{cc} \pi_{L}\left(z_{t}^{e},n_{t-1}\right) & =\left(1-n_{t-1}\right)\left(R_{0}z_{t}^{e}+S_{0}\left(1-z_{t}^{e}\right)\right)+n_{t-1}\left(R_{1}z_{t}^{e}+S_{1}\left(1-z_{t}^{e}\right)\right)\\ \pi_{H}\left(z_{t}^{e},n_{t-1}\right) & =\left(1-n_{t-1}\right)\left(T_{0}z_{t}^{e}+P_{0}\left(1-z_{t}^{e}\right)\right)+n_{t-1}\left(T_{1}z_{t}^{e}+P_{1}\left(1-z_{t}^{e}\right)\right) \end{array}$$

where $z_{t}^{e}$ is the effective fraction of low-effort extraction in the population with $z_{t}^{e}=1$ corresponding to the case where all individuals follow the low-effort strategy and $z_{t}^{e}=0$ corresponding to the case where all individuals follow the high-effort strategy. This results in an expected payoff advantage of low-effort extraction of

(Equation 4) $$\pi_{L}-\pi_{H}=\left(1-n_{t-1}\right)\left(\delta_{L}^{0}z_{t}^{e}+\delta_{H}^{0}\left(1-z_{t}^{e}\right)\right)-n_{t-1}\left(\Delta_{L}^{1}z_{t}^{e}+\Delta_{H}^{1}\left(1-z_{t}^{e}\right)\right)$$

where payoff differences at the corners of the state space are defined by

(Equation 5) $$\Delta_{L}^{1}=\pi_{H}\left(1,1\right)-\pi_{L}\left(1,1\right)=T_{1}-R_{1}$$

(Equation 6) $$\Delta_{H}^{1}=\pi_{H}\left(0,1\right)-\pi_{L}\left(0,1\right)=P_{1}-S_{1}$$

(Equation 7) $$\delta_{L}^{0}=\pi_{L}\left(1,0\right)-\pi_{H}\left(1,0\right)=R_{0}-T_{0}$$

(Equation 8) $$\delta_{H}^{0}=\pi_{L}\left(0,0\right)-\pi_{H}\left(0,0\right)=S_{0}-P_{0}$$

where capital Δ’s denote the incentives to switch to the high effort strategy and the lowercase *δ*′s denote the incentives to switch to the low effort strategy. The superscripts correspond to the current environmental state and the subscripts denote the current strategy that the population follows.

When payoff advantage is positive, $\pi_{L}-\pi_{H}>0$ extracting with low effort generates greater payoffs and when $\pi_{L}-\pi_{H}<0$, extracting with high effort yields higher payoffs.

#### Probabilistic strategy selection

Agents use a probabilistic decision rule to select their strategy, incorporating bounded rationality and uncertainty due to limited information.

• **Decision Function**: The probability of choosing high-effort extraction is given by a logistic function:

$$P_{H}=\frac{1}{1+\exp\;\left(-\frac{\sqrt{d_{i}}}{\sigma }\left(\pi_{L}-\pi_{H}\right)\right)}$$

• where:
  ○ $d_{i}$ is the degree (number of connections) of agent *i*, reflecting their visibility and confidence in their information.
  ○ *σ* is the slope parameter controlling the sensitivity of the decision to the payoff difference.
• **Strategy Determination**: Agents draw a random number from a uniform distribution to decide whether to adopt high-effort or low-effort extraction based on $P_{H}$.

#### Extraction and level of resource

The effective extraction of the population is based on a non-linear weighted average of all agents’ actions, with weights

(Equation 9) $$W_{i}=\frac{d_{i}^{\nu }}{\sum_{j}^{P}d_{j}^{\nu }}$$

that are determined by the network degrees *d* of all agents and the inequality of impact parameter *ν* so that when ν=0 all individuals have the same ability to impact the environment regardless of how many network connections they have and when ν>0 then the actions of more connected agents has disproportionate impacts on the dynamics of the environment.

These weights are combined with a strategy indicator variable, $S_{i}=\left\{0,1\right\}$, for each agent to compute the effective fraction of low extraction effort of the population as

(Equation 10) $$z_{t}=\sum_{i}^{P}S_{i}W_{i}$$

where, $S_{i}=1$ if agent *i* extracts with low effort and 0 if they extract with high effort.

Finally, we define the dynamics of the resource as

(Equation 11) $$n_{t}=n_{t-1}+\frac{\epsilon }{P}\left(z_{t}-n_{t-1}\right)$$

so that as a greater effective fraction of the population follow the low effort strategy, the environmental state increases and when the high effort strategy predominates, the environmental state declines. The law of motion dictates that the resource state $n_{t}$ closes a share $\frac{\epsilon }{P}$ of the gap between its current state $n_{t-1}$ and the effective normalized effort level $z_{t}$ in every period. The parameter ϵ denotes the relative speed of environmental and strategic dynamics. This law of motion captures the qualitative dynamics of decaying and renewing resources.^13^

#### Analytical limits

In this article, we recur to agent-based simulation due to two main factors. The first is that agents generate expectations about others, and we rely on a solid empirically tested literature of expectation formation. Agents switch between heuristics such they they provide better forecasts, and these heuristics coevolve with each other and the resource. Thus, agents’ extraction behavior and behavior change are not directly described by the number of high or low extractors (i.e., the time-indexed set (*z*,*n*) is not a Markov process). The second reason is that agents are placed on heterogeneous networks, restricting the information they receive about others. This creates further complications for analytical traceability. Both expectation formation and network heterogeneity are core elements of our research question.

This said, the general dynamics can be understood recurring to two foundational models. The first is that in Tilman et al. (2020),^13^ which follows a replicator dynamics for behavioral change. The replicator dynamics is the limiting case of various processes in which selection is asynchronous (one individual at a time) and biased towards a trait with higher fitness,^59^ and it occurs in a well-mixed population where all individuals are equally likely to interact with each other, receiving direct payoffs from those interactions. Those circumstances result in:

(Equation 12) $$\dot{z}=x\left(1-z\right)\left(\pi_{L}\left(z,n\right)-\pi_{H}\left(z,n\right)\right)\text{,}$$

(Equation 13) $$\dot{n}=\epsilon \alpha \left(z-n\right)\text{.}$$

The equation for $\dot{z}$ contains the payoff difference used in Equation 4 and the equation for $\dot{n}$ matches Equation 11 but in continuous time. This system can exhibit various types of dynamics.^13^ As we mentioned above, we consider the case where the incentives for this dynamics lead to a stable internal equilibrium with a mix of high and low extractors.

The second extension of this original model that brings us closer to the model described in this paper is that of considering agents that can create expectations. In another model by Tilman et al. (2023) (tilman_evolution_2023), individuals with different estimations of the resource co-evolve. While the equations are fairly similar, they now include (besides differential costs, which we here ignore for different heuristics) the number of high and low extractors for each of the heuristics. In that paper, the heuristics considered are linear extrapolation (which correspond to our heuristic number 2 with $\beta_{2}=1$). Further, in that paper, the change in heuristics is given by how much fitness it provides as opposed to how good it is at predicting the environment as we consider here.

In our work, we compensate this added complexity by using empirically grounded values for these heuristics.

#### Parameter values

The parameter values used in the simulations are provided by the Table *Parameter configuration of the model*. The four payoff advantage parameter values were chosen to correspond to a case from analytically tractable eco-evolutionary game studies where there is a stable estnal equilibrium in a continuous time, large population, well mixed version of the model where strategy dynamics are governed by the replicator equation, as analyzed in Tilman et al.^13^ Given this analytical result from a similar model, which holds regardless of the relative speed of environmental and strategy dynamics, we are able to isolate the impact of network structure and decision making heuristics on the joint dynamics of strategies and the environment in this paper. We further tested the impact of changing.

**Table:** Parameter configuration of the model

Parameter	Value	Description
$\delta_{H}^{0}=S_{0}-P_{0}$	−0.3	Payoff advantage of being an early adopter of low
		effort in a depleted environment
$\delta_{L}^{0}=R_{0}-T_{0}$	0.05	Payoff advantage of being a late adopter of low
		effort in a depleted environment
$\Delta_{L}^{1}=T_{1}-R_{1}$	0.7	Payoff advantage of being an early adopter of high
		effort in a bountiful environment
$\Delta_{H}^{1}=P_{1}-S_{1}$	0.45	Payoff advantage of being a late adopter of high
		effort in a bountiful environment
ϵ	0.25	Relative speed of environmental and strategic
		dynamics
*P*	1000	Number of agents
*σ*	0.1	Slope of sigmoid
$\beta_{1}$	0.63	Coefficient adaptive expectations
$\beta_{2}$	0.44	Coefficient trend-chasing expectations
$\beta_{3}$	−0.44	Coefficient contrarian expectations
*ρ*	0.9	Probability to update heuristic
*η*	0.7	Memory of prior heuristic utility
*ϕ*	100	Choice intensity
*λ*	250	Link density in the Barabasi-Albert algorithm
*ν*	0	Inequality of environmental impact

#### Alternative parameterization of payoffs

In this section, we introduce an alternative parameterization of the payoff functions that is mathematically equivalent but differs in how it is parameterized and in the interpretation of the parameters.

(Equation 14) $$\begin{array}{cc} \pi_{t}^{L}\left(n_{t-1},z_{t}^{e}\right) & =\alpha_{L}+\gamma_{1}^{L}z_{t}^{e}+\gamma_{2}^{L}n_{t-1}+\gamma_{3}^{L}n_{t-1}z_{t}^{e}\\ \pi_{t}^{H}\left(n_{t-1},z_{t}^{e}\right) & =\alpha_{H}+\gamma_{1}^{H}z_{t}^{e}+\gamma_{2}^{H}n_{t-1}+\gamma_{3}^{H}n_{t-1}z_{t}^{e} \end{array}$$

**Table:** Alternative equivalent parameter configuration of the model

Parameter	Value	Description
$\alpha_{L}-\alpha_{H}$	−0.3	Payoff advantage of being an early adopter of low
		effort in a depleted environment
$\gamma_{1}^{L}$	0.5	Linear relation of the average action other of users
		in the low-effort payoff
$\gamma_{2}^{L}$	1.5	Linear relation of the environment in the low-effort
		payoff
$\gamma_{3}^{L}$	0	Nonlinearity of low-effort payoff surface
$\gamma_{1}^{H}$	0.75	Linear relation of the average action of other users
		in the high-effort payoff
$\gamma_{2}^{H}$	2.25	Linear relation of the environment in the high-effort
		payoff
$\gamma_{3}^{H}$	0	Nonlinearity of high-effort payoff surface

**Table:** Mapping between alternative parameter configurations

Parameter	Description
$\delta_{H}^{0}=\alpha_{L}-\alpha_{H}$	Payoff advantage of being an early adopter of low
	effort in a depleted environment
$\delta_{L}^{0}=\alpha_{L}-\alpha_{H}+\gamma_{1}^{L}-\gamma_{1}^{H}$	Payoff advantage of being a late adopter of low
	effort in a depleted environment
$\Delta_{L}^{1}=\alpha_{H}-\alpha_{L}+\gamma_{1}^{H}-\gamma_{1}^{L}+\gamma_{2}^{H}-\gamma_{2}^{L}+\gamma_{3}^{H}-\gamma_{3}^{L}$	Payoff advantage of being an early adopter of high
	effort in a bountiful environment
$\Delta_{H}^{1}=\alpha_{H}-\alpha_{L}+\gamma_{2}^{H}-\gamma_{2}^{L}$	Payoff advantage of being a late adopter of high
	effort in a bountiful environment
$\alpha_{H}=P_{0}$	
$\alpha_{L}=S_{0}$	
$\gamma_{1}^{L}=R_{0}-S_{0}$	
$\gamma_{2}^{L}=S_{1}-S_{0}$	
$\gamma_{3}^{L}=R_{1}-R_{0}+S_{0}-S_{1}$	
$\gamma_{1}^{H}=T_{0}-P_{0}$	
$\gamma_{2}^{H}=P_{1}-P_{0}$	
$\gamma_{3}^{H}=P_{0}-P_{1}+T_{1}-T_{0}$	

### Quantification and statistical analysis

The results in Figure 2 are based on 100 simulations. The results in Figures 3 and 4 are based on a single simulation. The results in Figure 5 are based on 30 simulations per level of skewness of the degree distribution. The results in Figure 6 are based on 30 simulations per level of inequality in environmental impact.
